# Nexus Advances Using Marine Biopolymeric Gel Material as a Photocatalyst for the Oxidation of Agricultural Wastewater Containing Insecticides

**DOI:** 10.3390/gels9110864

**Published:** 2023-10-30

**Authors:** Ehssan Ahmed Hassan, Maha A. Tony

**Affiliations:** 1 Department of Biology, College of Science and Humanities, Prince Sattam bin Abdul Aziz University, Al-Kharj 11942, Saudi Arabia; 2 Department of Zoology, Faculty of Science, Suez Canal University, Ismailia 41522, Egypt; 3 Basic Engineering Science Department, Faculty of Engineering, Menoufia University, Shebin El-Kom 32511, Egypt; 4 Advanced Materials/Solar Energy and Environmental Sustainability (AMSEES) Laboratory, Faculty of Engineering, Menoufia University, Shebin El-Kom 32511, Egypt

**Keywords:** methomyl wastewater, chitosan biopolymer, photocatalyst, nanopowders, gel

## Abstract

The attention of the research community is focused not only on waste elimination, but also on waste valorization. The natural marine biopolymer gel substance chitosan, which can be derived from the waste substances of marine life, is a polymer-matrix-based nanocomposite. Chitosan attracts special attention due to its potential applications, especially in wastewater treatment. In this regard, magnetite-incorporated chitosan powders of nanometer scale were synthesized by a simple co-precipitation method to attain the dual functions of chitosan gel and magnetite. The synthesized magnetite-incorporated chitosan nanopowders were verified using X-ray diffraction (XRD), Fourier-transform infrared (FTIR) spectroscopy, a vibrating-sample magnetometer (VSM), a scanning electron microscope (SEM), and transmission electron microscopy (TEM) images, which showed that the synthesized magnetite-incorporated chitosan was nanosized. The superior application of such a material to offset the deterioration of the environment caused by insecticides is attained through a photocatalytic reaction. The experimental results verified the function of magnetite-incorporated chitosan, since it increased the composite-specific surface area, resulting in high methomyl molecule oxidation. Methomyl oxidation reached almost complete insecticide removal (99%) within only one hour of irradiance time. The optimal operational conditions were investigated, and the maximal removal rate occurred when the aqueous solution was at an acidic pH of 3.0. The reaction was affected by differing hydrogen peroxide and catalyst doses, and the optimized reagent was recorded at the levels of 40 and 400 mg/L of catalyst and hydrogen peroxide, respectively. Also, catalyst reusability was attained, confirming its sustainability, since it could be used for successive cycles. From the current investigation, it is proposed that magnetite–chitosan nanoparticles could serve as a promising photocatalyst for the elimination of insecticides from wastewater in a green manner.

## 1. Introduction

Recently, the ever-increasing significance of polymeric nanocomposites, in which nanomaterials are embedded in a polymer matrix, has attained great attention due to their potential application in numerous fields of science and technology [1,2]. Additionally, environmentally friendly magnetite nanoparticles are regarded as important, due to their abilities of environmental remediation and toxicological elimination [3]. This is a compelling reason to probe novel routes for combining these two materials for wastewater treatments. At the end of the 18th century, Charles Hatchett declared chitin to be a gel biopolymer [4]. However, its real applications only originated in the 19th century. Chitosan is a derivative of chitin; it was this conversion that attracted both academic and industrial attention for its wide applications [5,6,7]. Furthermore, the incorporation of chitosan with magnetite, to acquire a multifunctional chitosan–magnetite composite, opens up new avenues to attain sustainable applications [8]. The reliable characteristics of magnetite, including its magnetic nature and its optical and electronic characteristics, make it a good candidate to combine with the chitosan gel biopolymer [9,10,11]. One of the attractive features of such a chitosan–magnetite composite is its superparamagnetic performance, which confirms the catalyst to be sustainable, recoverable, and recyclable. This chitosan–magnetite composite has been previously applied to eliminate various types of dyes from aqueous media; so far, it has not been applied to eliminate insecticides from wastewater, according to the literature cited [12,13,14].

Green wastewater implementation, using natural resources and environmentally friendly materials, is an important topic. Advanced oxidation processes (AOPs), using green materials as powerful oxidants, have showed complete mineralization of pollutants [8]. AOPs are signified as a prerequisite for safe wastewater disposal facilities [15,16]. However, further studies should be conducted to validate their potential economic applications. Liu et al. [13] combined ozone with ultraviolet light as a source of advanced oxidation that also used hydrogen peroxide. The Fenton reaction is categorized as one of the fundamental features of AOPs, as a homogeneous process. While this process has shown significant wastewater elimination results, current research interests involve searching for a novel Fenton technique, aimed at avoiding the current system’s limitations [9,17]. These limitations include an acidic working pH range, resultant sludge residue from the treatment, and the caustic chemicals applied [18,19,20]. Hence, developing a reusable catalyst could be more cost-effective and overcome these limitations to become a sustainable catalytic treatment. Moreover, the use of natural resources is a promising technique to attain such criteria as an opportunity for eco-friendly oxidation. Furthermore, updated studies have improved the oxidation of the Fenton reaction using nanoparticles [14].

The contamination of water by toxic effluents released from the industrial sector represents a risk to both the environment and human health [21]. Due to various organic and inorganic materials, as well as pathogenic organisms, contained in such wastewater discharge, it is signified as a toxic effluent. Thus, direct discharge into the ecosystem causes severe damage to water bodies [22,23]. In this regard, treating the effluents is a must. Various efforts based on conventional water treatment techniques, including physical, chemical, and biochemical treatments, have been introduced [24]. However, recently, more efforts have been made to be sustainable, which could not be satisfied by traditional treatments such as membrane systems, adsorption facilities, reverse osmosis, or biochemical treatments [25,26,27]. This is due to these techniques not being able to attain complete treatment, along with their lack of recyclability, which limits their applications [28,29]. Thus, the emergence of AOPs, which possess a high conversion rate when eliminating toxic substances [30,31], has attracted researchers in the field of wastewater treatment, who wish to use sustainable materials [32,33].

In line with the principles of green chemistry, chitosan, a biopolymeric material, was incorporated with magnetite to form a composite substance, using a simple co-precipitation methodology. Then, the prepared samples were characterized using X-ray diffraction (XRD), Fourier-transform infrared spectroscopy (FTIR), vibrating-sample magnetometer (VSM), scanning electron microscope (SEM), and transmission electron microscopy (TEM) images, which verified the presence of composite nanoparticles. Subsequently, to overcome the global challenges of wastewater treatment and its negative effect on the water quality, the use of such a composite for eliminating wastewater deterioration was introduced. The system was applied for oxidizing the insecticide methomyl using an ultraviolet Fenton reaction. The system parameters were studied, the optimized conditions were recorded, and the results confirmed the efficiency of the system as a novel treatment for agricultural wastewater.

## 2. Results and Discussion

### 2.1. Characterization of the Composite Material

#### 2.1.1. XRD Analysis

**Figure 1:**
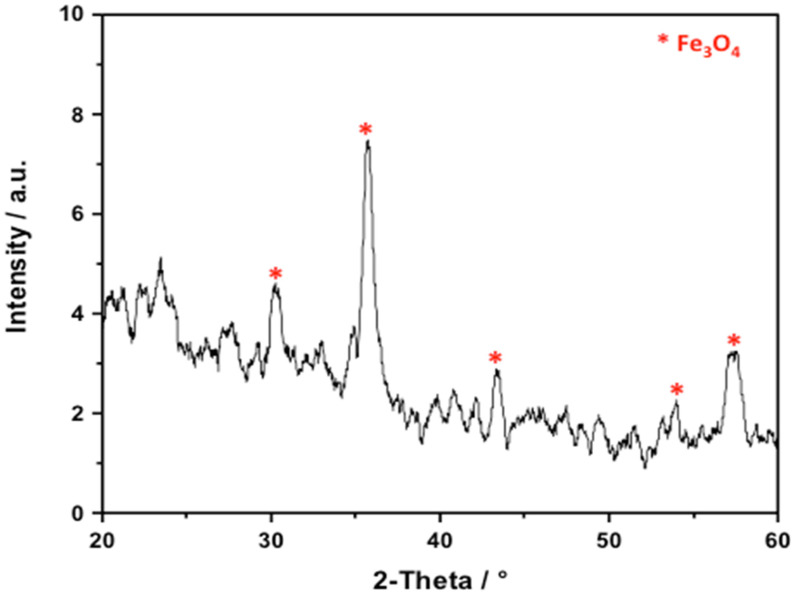
XRD pattern of the chitosan–magnetite nanocomposite.

The X-ray diffraction (XRD) pattern of the synthesized composite is displayed in Figure 1. As shown in Figure 1, strong diffraction peaks arose at diffraction angles of 2θ = 30.1, 35.6, and 43.2°, which reflect the peaks of the [220], [311], and [400] planes of magnetite [26,33]. According to the XRD graph, these magnetite nanoparticles were crystalline in nature and coated the amorphous chitosan. Also, it should be noted that the well-defined sharp diffraction peaks and the broadening of those peaks shows the small size of these nanosized particles without producing damage to the magnetite crystal structure.

#### 2.1.2. SEM Micrographs

**Figure 2:**
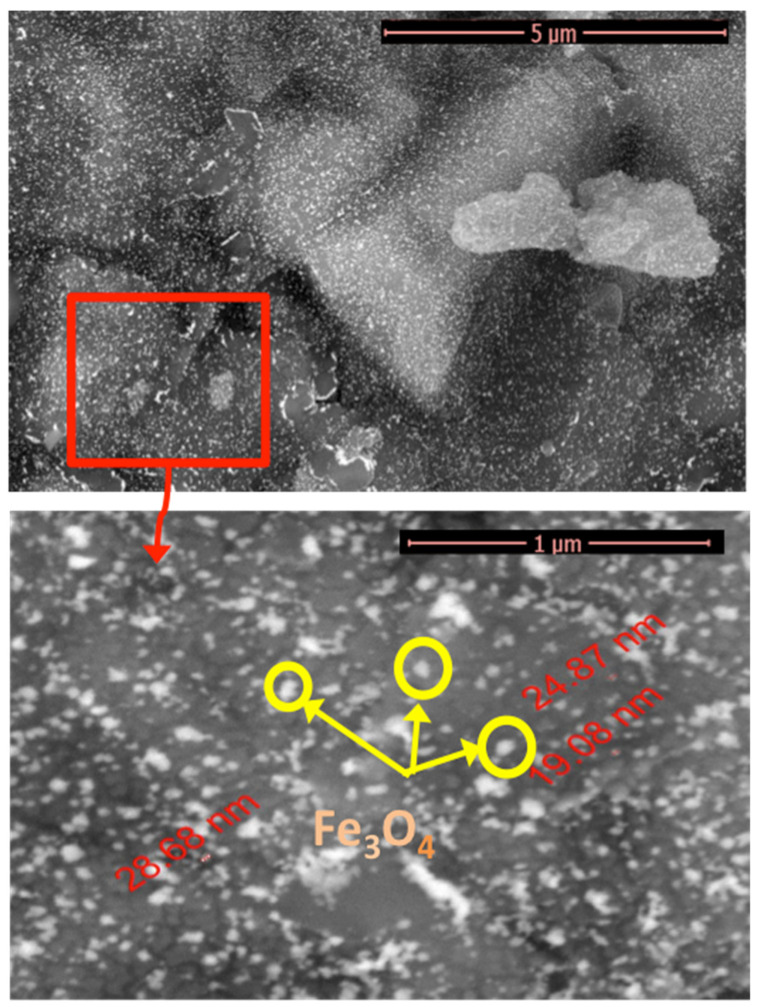
SEM images of the chitosan–magnetite composite material at different magnifications.

The characteristic morphology of the chitosan–magnetite composite, as a photocatalyst, was explored by scanning electron microscope (SEM) analysis, and the images at different magnifications are shown in Figure 2. According to the images, agglomerated nanoparticles were found at the surface of the chitosan. Chitosan plays a vital role as a crosslinker to establish a more spherical morphology of the nanoparticles. Magnetite nanoparticles are shown as spherical particles with a narrow grain-size distribution, representing the magnetite nanoparticles as displayed in the inset of the Figure 2, with the smooth surface of chitosan. Crosslinked polymers can adsorb and retain large amounts of water. Thus, such polymer matrices adsorb water and pollutants through diffusion mechanisms and macromolecular relaxation during swelling processes. Therefore, such polymers are suitable candidates for the treatment of polluted water, due to their superior properties of high chemical stability, low economic cost, and the accessibility of recovery. Due to the presence of large numbers of hydroxyl and amino groups, chitosan can easily adsorb various pollutants. Furthermore, chitosan’s properties can be enhanced by adding nanoparticles, or by crosslinking with synthetic polymers or biopolymers.

#### 2.1.3. TEM Images

**Figure 3:**
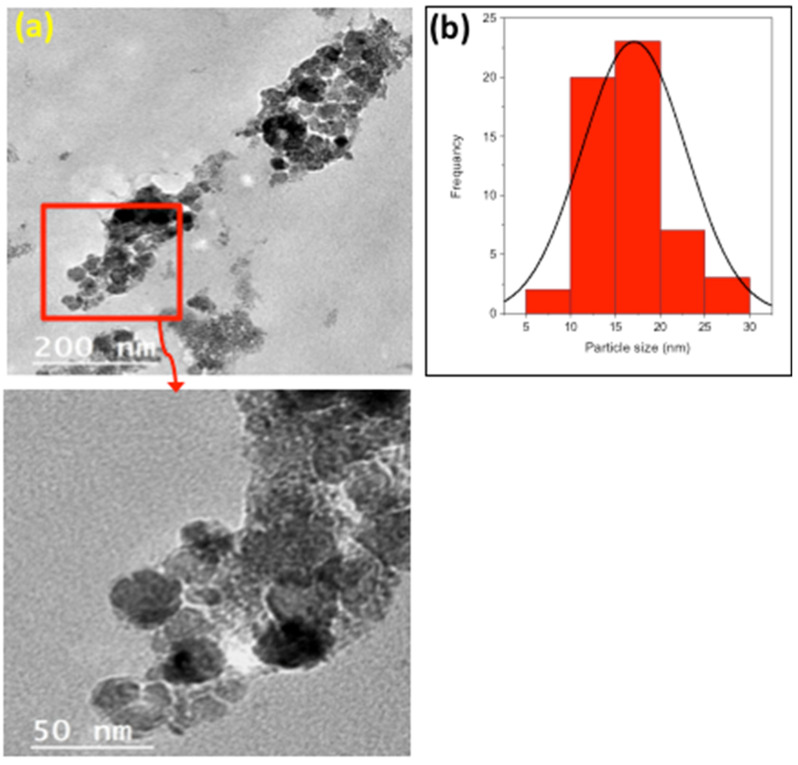
TEM images of the chitosan–magnetite composite material at different magnifications. (**a**) Transmission electron microscope (TEM) analysis. (**b**) The particle-size distribution of the chitosan–magnetite composite.

In order to illustrate and clarify the morphology of chitosan decorated with magnetite nanoparticles as a composite, transmission electron microscope (TEM) analysis was conducted, and the images are displayed in in Figure 3 at different magnifications. The images exhibit that the chitosan is decorated with magnetite nanoparticles with a spherical shape. The chitosan material was loaded with a homogeneous distribution of spherical magnetite spheres. However, it is noteworthy that due to the dipole–dipole attraction forces linked to the magnetite particles, some of the nanoparticles aggregated with one another. In summary, the TEM images of the composite sample reveal the homogeneity of the sample, which reflects the homogeneous dispersion of magnetite particles in the composite. It is noteworthy that such preparation methods are based on co-precipitation and the use of NaOH as a surfactant to control the semi-spherical shape, and the pH value is associated with the particle-size distribution.

Furthermore, the particle-size distribution of the chitosan–magnetite composite was calculated, and the histogram is displayed in the inset of Figure 3b. The particle-size distribution of the composite ranged from 5.22 to 30.26 nm, with an average particle size of 17.35 nm. These results confirm the nanosized range of the synthesized composite. Such particle sizes are quite reasonable to offer a high surface area of the particles to enable an efficient photocatalytic degradation reaction. Commonly, particle size has an effect on the treatment, since it affects the surface area of the catalyst responsible for the pollutant oxidation. Hence, a low particle size range is favorable, since such particles possess a high surface area and, therefore, the number of active sites available for treatment is high.

#### 2.1.4. FTIR Analysis

**Figure 4:**
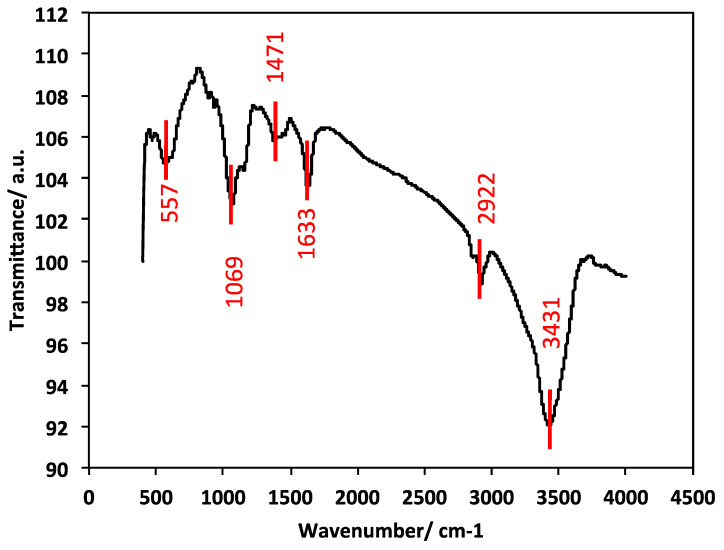
FTIR spectrum of the chitosan–magnetite composite material.

Fourier-transform infrared (FTIR) spectroscopy of the prepared chitosan–magnetite composite was conducted to confirm the encapsulation of the magnetite nanoparticles on the chitosan, as well as the formation of the composite. The data displayed in Figure 4 show the spectra between 400 and 4000 cm^−1^. The absorption bands at 3431 cm^−1^ due to −NH or –OH stretching vibrations signify the characteristic IR spectrum of chitosan, while those at 2922 and 1471 cm^−1^ represent the –CH stretching of the copolymer of chitosan [32]. The amide II bands (−NH bending and C=N stretching) appeared at 1633 and 1469 cm^−1^, respectively, for the presence of chitosan [8]. The characteristic peak at 557 cm^−1^ reflects the Fe−O stretching vibration, which represents the stretching vibration of magnetite nanoparticles [33]. Also, the peak of −CH stretching was confirmed at 2922 cm^−1^ [33]. Additionally, the C-O-C group is represented by the band at 1069 cm^−1^.

#### 2.1.5. VSM Analysis

**Figure 5:**
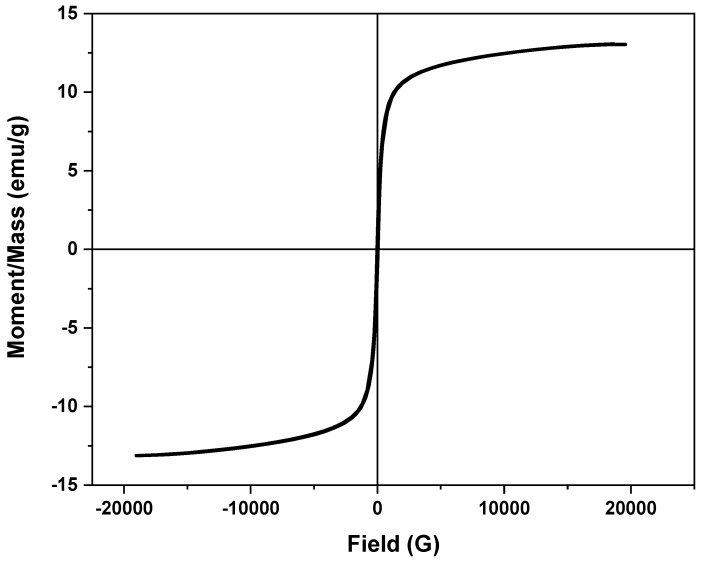
VSM measurement of the chitosan–magnetite composite material.

VSM is still signified as efficient way to evaluate substances’ magnetism. This is assessed as follows: the larger the hysteresis loop, the better the magnetic susceptibility [32]. Using this concept, the saturation magnetization of the attained chitosan–magnetite sample was investigated, and the data are exhibited in Figure 5. The saturation magnetization of the prepared sample was 13.12 emu/g, indicating the presence of magnetism even though the chitosan was conjugated with the magnetite particles in the composite. However, according to the literature, the saturation magnetization of pristine magnetite nanoparticles is 69.48 emu/g [32], which is a lot higher than that of the proposed composite. But it is noteworthy that the sample still possessed a magnetic characterization and could be signified as a good superparamagnetic material. Such characteristics indicate that the material possesses the merits of easy solid–liquid phase separation after the wastewater treatment. Thus, the catalyst is signified as sustainable, since it is recyclable for successive treatments.

### 2.2. Studies on Methomyl Oxidation

#### 2.2.1. Effects of Reaction Time and Methomyl Loading

**Figure 6:**
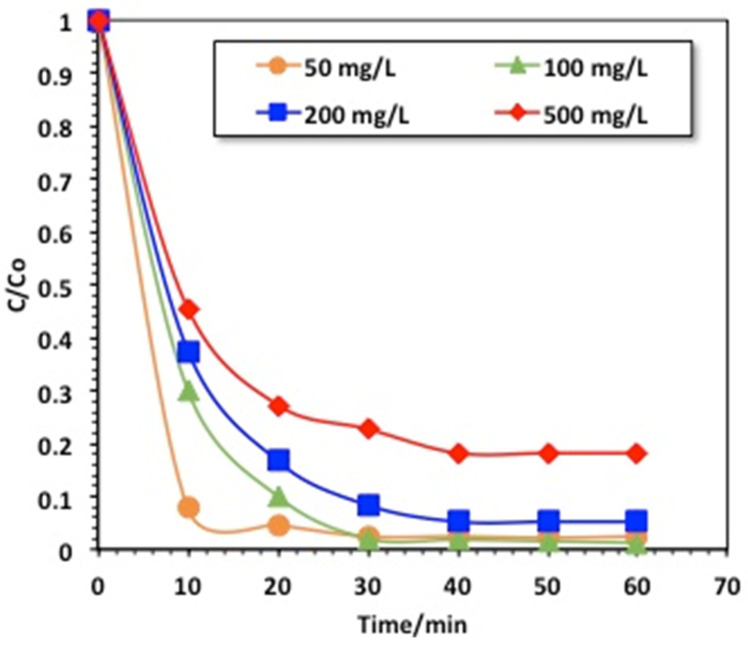
Effects of reaction time and methomyl loading on the chitosan–magnetite-based oxidative system.

In order to reach a real-scale application where the initial pollutant concentration was altered, the methomyl wastewater loading was achieved as a function of reaction time. Figure 6 jointly displays the reaction time of oxidation and the effect of the methomyl loading using the chitosan–magnetite composite for the catalytic oxidation of a modified Fenton system under UV illumination. The efficiency of such performances was evaluated for various methomyl concentrations under oxidation with H_2_O_2_/chitosan–magnetite of 40 and 100 mg/L, respectively, at pH 3.0.

The results displayed in Figure 6 exhibit that even though all of the methomyl loading could be oxidized by the H_2_O_2_/chitosan–magnetite-based Fenton reaction oxidation protocols, the removal efficiency differed according to the methomyl load, where higher loading reduced the efficiency to only 80%, compared to complete removal within 60 min for the low concentrations (50 mg/L). To illustrate, hydroxyl radicals were traced through the catalytic decomposition of the H_2_O_2_ reagent by the catalyst nanocomposite material. This could be attributed to the fact that the hydrogen peroxide and catalyst were the same, while the methomyl loading was increased, meaning that the OH radicals generated were not sufficient to oxidize all of the methomyl molecules. Also, it is noteworthy that higher removal efficiency was attained within the initial reaction time for all of the methomyl concentration systems. Additionally, whereas chitosan was previously signified just as an adsorbent substance, scattered literature [8,34] has proposed it as a photocatalyst due to its photocatalytic activity. Additionally, the available active vacant adsorbent sites on the chitosan–magnetite composite are not enough to adsorb methomyl molecules [33]. Hence, chitosan–magnetite is proposed as a recoverable photocatalyst that verifies the merits of sustainability. The trend of increasing the methomyl loading resulted in a decline in the oxidation efficacy, in accordance with the previously reported literature on treating dye-polluted water [8].

#### 2.2.2. Effect of Hydrogen Peroxide

**Figure 7:**
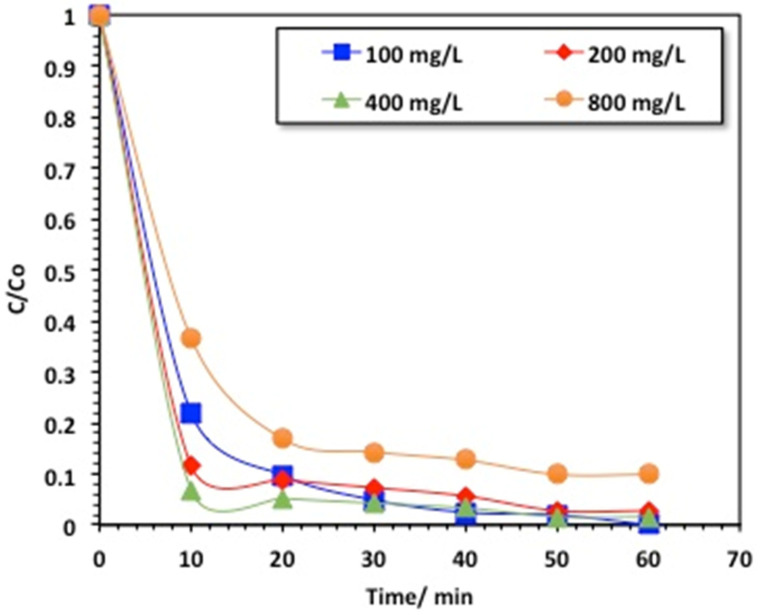
Effect of hydrogen peroxide loading on the chitosan–magnetite-based oxidative system.

In the light of attaining the highest composite activity, it is essential to achieve the optimal concentration of hydrogen peroxide incorporated into the magnetite-decorated chitosan biopolymer. This leads to the initiation of the Fenton reaction with the supplemented optimal dose of H_2_O_2_. Therefore, the hydroxyl radicals generated can reach the maximal value. In this regard, initially, the hydrogen peroxide was combined with chitosan–magnetite at concentrations varying from 100 to 800 mg/L (under acidic conditions (pH 3.0) and with a catalyst dose of 40 mg/L), and the methomyl removal efficiency was recorded. The results are exhibited in Figure 7, demonstrating that the increase in the hydrogen peroxide dose from 100 to 400 mg/L could greatly affect the oxidation efficiency. However, an opposite trend was found when the dose reached 800 mg/L. This result could be associated with the presence of OH radicals, which are the main drivers of oxidation. The maximal occurrence of OH radicals is related to the optimal quantities of hydrogen peroxide and catalyst. The result leads to an increase in the photocatalytic methomyl removal efficiency. Also, the photocatalytic activity is highest when the hydrogen peroxide concentration is 400 mg/L, resulting in an increased oxidation yield [35].

#### 2.2.3. Effect of Chitosan–Magnetite Nanocomposite Loading

**Figure 8:**
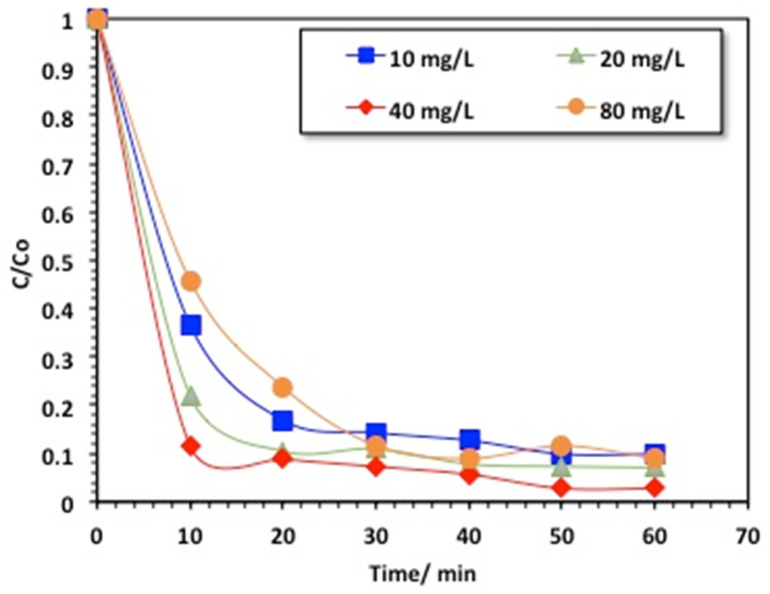
Effect of catalyst loading on the chitosan–magnetite-based oxidative system.

In an effort to achieve optimized catalyst use for appropriate applications, the effect of the catalyst (chitosan–magnetite) loading on the performance of the photocatalytic oxidation was tested. In this regard, the loading of the chitosan–magnetite nanocomposite was checked to investigate the oxidation capabilities of H_2_O_2_/chitosan–magnetite (Figure 8). The data exhibited in Figure 8 display the effects of the Fenton oxidation on different chitosan–magnetite loadings, from 10 to 80 ppm; however, all other operating parameters were kept constant (400 mg/L for H_2_O_2_, and pH of 3.0). The maximal methomyl removal effectiveness was reached with a complete (99%) insecticide removal when 40 mg/L catalyst was used as a source of the Fenton reaction, whereas it reached 90, 91, and 97% removal within 60 min for chitosan–magnetite concentrations of 10, 20, and 80 mg/L, respectively. This might be due to the H_2_O_2_ dose of 400 mg/L, which was kept constant in all experiments. Hence, the attained yield of ^•^OH radicals generated was not sufficient to achieve complete methomyl removal. This means that the hydrogen peroxide should be in balance to maximize the hydroxyl radicals’ production and prevent the reduction in the effective amount of •OH radicals. A similar validation was previously recorded by [35] when treating wastewater via the photo-Fenton reaction test.

#### 2.2.4. Effect of pH on the Composite Performance

**Figure 9:**
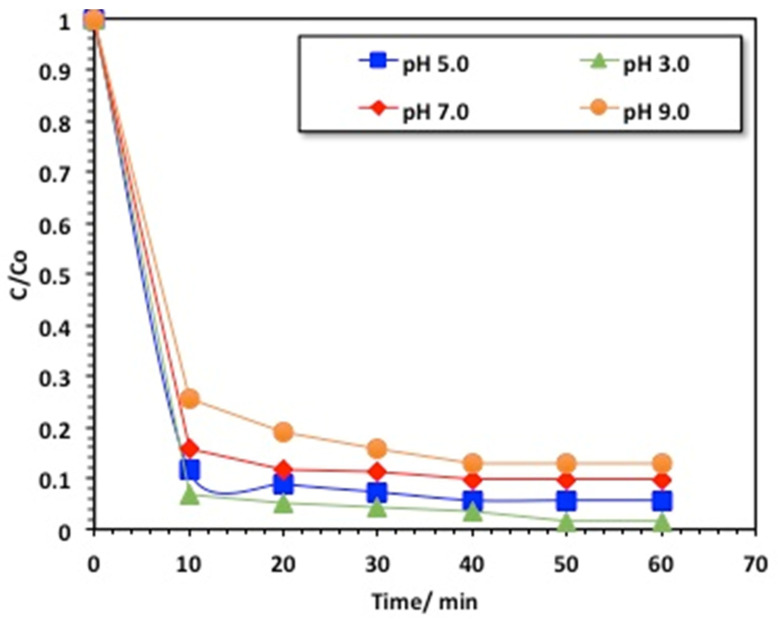
Effect of solution pH on the chitosan–magnetite-based oxidative system.

The effect of the initial solution pH on the methomyl oxidation was assessed through changing the pH in the range from 3.0 to 9.0, with 400 mg/L hydrogen peroxide and 40 mg/L chitosan–magnetite catalyst under UV irradiance. The data exhibited in Figure 9 reveal that the methomyl oxidation rate improved with the decline in the pH value, and the optimal operational pH value was in acidic conditions, corresponding to the value of 3.0. But increasing the pH value resulted in deterioration in the methomyl oxidation rate. Remarkably, alkaline pH values were critical, since they decayed the oxidation rate.

The effect of pH on the methomyl oxidation can be clarified by the surface charge of the chitosan–magnetite catalyst. According to the previous data cited, the point of zero charge of the chitosan–magnetite catalyst composite was recorded at pH 7.23 [8]. Hence, the surface of the chitosan–magnetite catalyst could be negatively or positively charged, due to dissociation of the basic character of the catalyst at a pH_PZC_ of 7.9. Therefore, the alteration in the medium’s pH imitates the surface charge of the catalyst and, hence, affects the photocatalytic and adsorption efficiencies. Consequently, this might be illustrated by the presence of low electrostatic interactions between the methomyl molecules and the chitosan–magnetite catalyst at alkaline pH, hence reducing the oxidation rate is [35].

#### 2.2.5. Temperature Effect, Oxidation Kinetics, and Thermodynamic Determination

**Figure 10:**
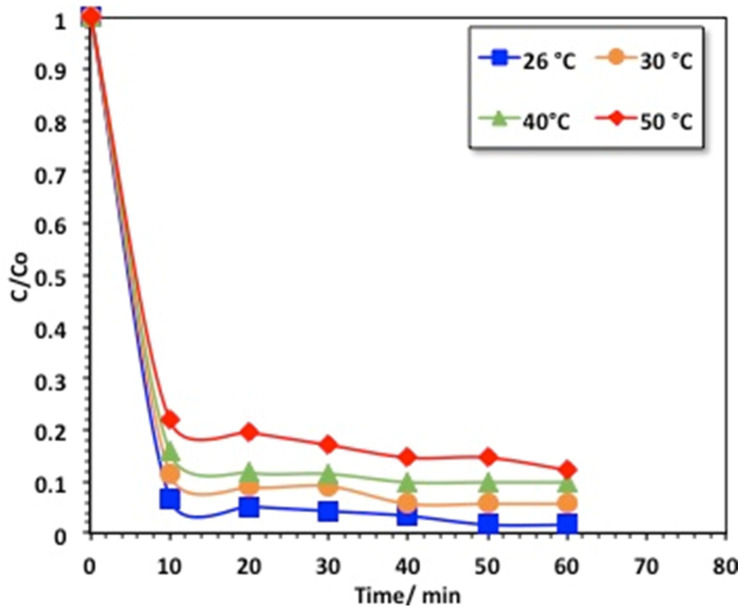
Effect of temperature on methomyl removal by the chitosan–magnetite system.

It is essential to demonstrate the effect of temperature for “real practical applications”, since it affects reaction rates. Also, in real life, the discharged aqueous effluent may be at various temperatures. With this in mind, the aqueous methomyl solution’s temperature was varied from 26 to 60 °C to assess the effect of temperature on the oxidation reaction. The experimental data displayed in Figure 10 demonstrate a reduction in the methomyl oxidation rate with the elevation of the solution temperature in the studied range. Almost complete (99%) methomyl removal was attained in about 60 min of reaction time at room temperature, but at higher temperatures (i.e., above room temperature (26 °C)) the efficiency declined. It is noteworthy that the optimal temperature corresponded to 26 °C for achieving better oxidation, in accordance with the previous work reported in [36] on treating landfill leachate through the Fenton reaction. Nevertheless, the overall yield of hydroxyl radicals is reduced at high temperatures, further reducing the methomyl oxidation. Also, a high aqueous solution temperature decomposes hydrogen peroxide into O_2_ and H_2_O; hence, a reduction in the overall oxidation is also achieved. However, scattered works have found that high temperatures enhance the OH radicals’ generation and, thus, enhance the oxidation rate [37,38,39]. Additionally, it is notable that some other researchers have specified that temperature has a small terminal effect on the Fenton system compared to the abovementioned parameters.

The oxidation kinetics is useful for the estimation for the reactor design and the system control, which both affect the process’s cost. Kinetic studies of oxidation are essential for the heterogeneous combination of a chitosan–magnetite catalyst with hydrogen peroxide to eliminate and oxidize methomyl in an aqueous matrix and check the complexity of intermediates formed during the Fenton-based system. For further examining the oxidation test through the chitosan–magnetite Fenton system, a kinetics study was conducted. Based on the overall methomyl content, the kinetics study was carried out, the methomyl oxidation was examined as a function of time, and the data were fitted in linearized integrated equations for both first- and second-order kinetics models (as exhibited in Table 1).

The rate constants were then investigated by calculations based on linearized equations (Table 1). The correlation coefficient (*R*^2^) values were used to determine the best-fitting model. High values of the correlation coefficient represent the goodness of fit of a model. We compared the *R*^2^ values estimated from the aforementioned equations to suggest the best-fitting model. Thus, oxidation followed the second-order reaction kinetics, since the correlation coefficient values were the highest (0.86–0.94) in comparison to the first-order reaction kinetics model. Correspondingly, the reaction time half-life (*t*_1/2_) values were also calculated using half-life equations derived for first- and second-order kinetics (the results are tabulated in Table 1). It was determined that the *t*_1/2_ values calculated through the second-order kinetics equation were closely linked to the experimental data. This estimation was in accordance with the previous cited data in research articles [39,40,41].

**Figure 11:**
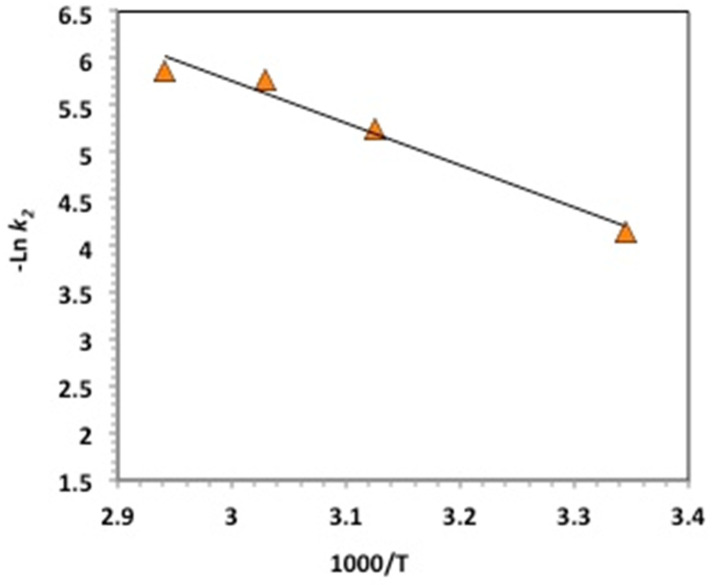
Arrhenius plot based on pseudo-second-order kinetics constants.

To obtain a good overall understanding of the reaction system, it is essential to further understand the effect of temperature on the reaction’s thermodynamics. The thermodynamic activation values of the parameters were assessed via the Arrhenius fit ($lnk_{2}=lnA-\frac{E_{a}}{RT}$), which is based on the second-order kinetics model, since it fitted the experimental data well, where *A* and R are a pre-exponential factor and the gas constant, respectively, while *Ea* is the energy of activation, which is determined from this relation by plotting *lnk_S_* versus 1/T, giving a linear relation (Figure 11) whose slope is matching (−*Ea/R*). Then, the Eyring equation ($k_{2}=\frac{k_{B}T}{h}e^{\left(-\frac{∆G^{\prime }}{RT}\right)}$) (*k_B_* and *h* are Boltzmann’s and Planck’s constants, respectively) was used to evaluate the thermodynamic parameters. Enthalpy (∆*H*′) and the entropy (∆*S*′) of activation can be estimated from ($∆H^{\prime }=E_{a}-RT$) and ($∆S^{\prime }=(∆H^{\prime }-∆G^{\prime })/T$), respectively [42].

In summary, Table 2 shows the thermodynamic parameters, as well as the energy of activation. The data reveal that the oxidation proceeds in a non-spontaneous manner, since the Gibbs free energy of activation is positive, which means that the oxidation is endergonic. This non-spontaneity is verified by the negative values of entropy (∆*S*′) and positive values of the enthalpy of activation ($∆H^{\prime })$ through the whole temperature range. Negative values of ∆*S*′ were attained because of the increase in randomness of the methomyl molecules and the ·OH radical species generated [32]. The reaction is endothermic in nature ($∆H^{\prime }$> 0), and the ∆*S*′ decreased with the increase in temperature. This is consistent with the previous findings in the literature [8] on treating aqueous contaminants.

#### 2.2.6. Catalyst Stability Assays

**Figure 12:**
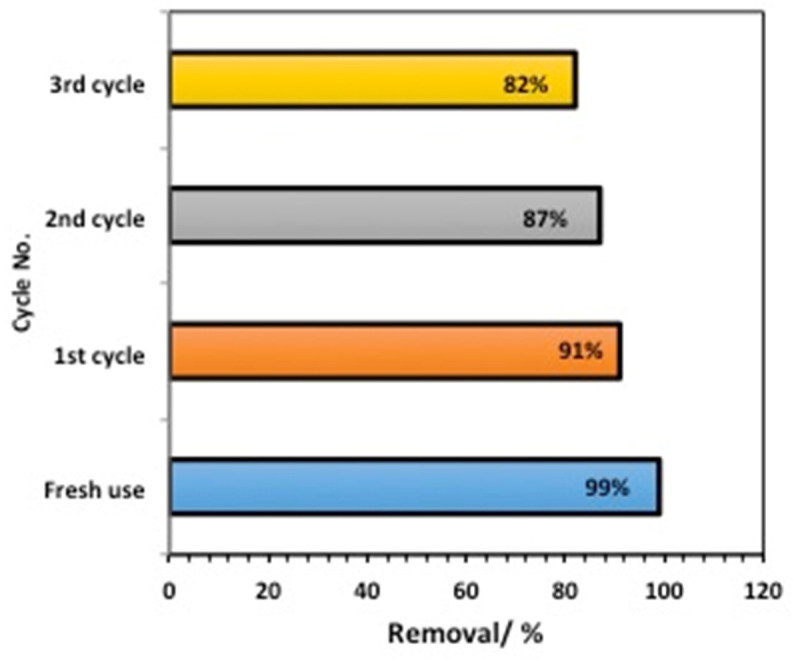
Reuse performance of the chitosan–magnetite composite catalyst.

To confirm the catalyst’s sustainability, catalyst recovery and reuse were evaluated. Initially, the catalyst was collected after fresh or successive use. The catalyst was recoverable and, thus, no expected byproducts were attained. The material was collected via filtration. Subsequently, it was washed with distilled water. Thereafter, the washed material was dried in an electric oven (150 °C) for one hour. With the same illumination time, the catalyst activity was checked using a catalytic Fenton reaction (Figure 12). A high removal efficiency of 91% was achieved after the first use, in comparison to 99% for fresh use. However, this declined to 82% after the next use, which is still considered to be a high removal efficiency. These results confirm the catalyst’s sustainability. However, the decline in the efficiency is linked to the insecticide’s occupation of the catalyst surface. This could be linked to the composite material’s active sites, which might be occupied by some organic intermediates, which shield those active centers and, thus, block them from attacking the organic pollutants. Consequently, the overall reaction rate was reduced [26].

#### 2.2.7. Comparative Investigation

Table 3 shows the Fenton oxidation based on the current environmentally benign investigation, compared with the findings of previously studies. Some previous studies cited in the literature were assessed and compared with the present data based on the results of the current work. The comparative data summarized in Table 3 are based on the comparison of previous composite-based Fenton oxidation systems with the current composite’s performance.

Mixed copper oxides have been investigated as Fenton source materials [43,44,45,46]; however, scattered authors [8,23,46] have used magnetite-based campsite as a Fenton source. Such magnetite-based campsite has been recorded as a superior catalyst, since it is an easily recoverable substance, offering the opportunity for reuse. Thus, such studies possess the advantage of using a recyclable material. Also, using a waste material in the composite to convert the catalytic oxidation is a win–win technology, such as in the current study, where we introduced chitosan from marine waste, as well as the works introduced in other research [33,44] that used aluminum-based sludge material. Not only is the use of catalysts from waste streams environmentally benign, it also minimizes the cost of the treatment. Thus, the current study combined catalyst reusability through a gel polymer “chitosan”-based composite and environmental sustainability by using chitosan waste material. A remarkably small amount of material was used in comparison to other studies, suggesting the significance of the current work’s composite as an economical and benign substance. In summary, it is essential to mention that complete pollutant oxidation was achieved in the current work.

**Table 3:** Comparative investigation between the current study and various composites from Fenton systems reported in the literature.

Composite Type	Induction Source of Fenton System	Pollutant	Pollutant Load	Catalyst Dose	pH	Oxidation (%)	Ref.
Chitosan–magnetite	Ultraviolet	Methomyl insecticide	50 ppm	3.0 g/L	3.0	Complete oxidation	Current work
Chitosan–magnetite	Ultraviolet	Basic blue dye	10 ppm	2.4 mg/L	7.0	Complete oxidation	[8]
LaFeO_3_/BiOBr	Ultraviolet	Rhodamine B dye	5 ppm	0.1 mg	Not available	98.2%	[14]
Mixed copper oxides	Microwave irradiance	Methomyl insecticide	50 ppm	3.0 g/L	6.5	91%	[43]
Silica-supported iron	Solar radiation	Methomyl insecticide	100 ppm	103 mg/L	2.8	98%	[44]
Magnetite–CeO_2-_g-C_3_N_4_	Visible light	Tetracycline hydrochloride	50 mg/L	50 mg/L	2.7	96.63%	[46]
Silver/bismuth/iron oxides	Visible light	Methyl orange	40 ppm	0.6 g/L	Not available	97%	[11]
Cellulose–magnetite	Ultraviolet	Red K-HL dye	50 ppm	1 g/L	3.0	99%	[45]
Alum sludge waste–magnetite	Ultraviolet	Methomyl insecticide	50 ppm	50 mg/L	6.0	Complete oxidation	[22]
Titanium/iron oxides	Ultraviolet light	Methyl orange	80 ppm	200 mg/L	4.5	97%	[34]
Aluminum-based waste–magnetite	Ultraviolet	Levafix blue dye	50 ppm	2 g/L	2.0	Complete oxidation	[23]
TiO_2_@NH_2_-MIL-88B(Fe)	Visible light	Methylene blue	100 ppm	200 mg/L	7.0	Complete oxidation	[34]

## 3. Conclusions

The current investigation effectively tailored and perceptively fabricated magnetic chitosan and converted it from a marine gel waste into a valuable photocatalyst through the co-precipitation method. The morphology and structure of the synthesized magnetite–chitosan substance were effectively investigated and studied. The photocatalytic experiments revealed that the Fenton-based chitosan-conjugated magnetite nanoparticles, as a source of the Fenton reaction, could completely oxidize methomyl in wastewater. The oxidation rate reached 99%, which is almost complete removal, within 1 h of irradiance time, which verifies the effective oxidation of methomyl molecules from contaminated water. Additionally, the operative parameters were optimized, and the kinetics of the reaction was examined. The kinetics data showed the reaction following the second-order reaction kinetics model, with an activation energy that reached 37.39 kJ/mol. Also, the catalyst was recovered for reuse, and its sustainability was verified, which confirmed the catalyst as an eco-friendly material from various perspectives. Hence, the proposed catalyst is a promising candidate for eliminating insecticides from agricultural effluent.

## 4. Materials and Methods

### 4.1. Synthesis of Chitosan–Magnetite Nanocomposite

Chitosan, as a marine polysaccharide derived from a marine creature, i.e., shrimp shell waste, was introduced as a gel biopolymeric material. Chitosan was applied in its dried form and decorated with magnetite to form a nanocomposite that was synthesized by co-precipitation, followed by the hydrothermal route [33]. Droplets of acetic acid were added to a definite weight of chitosan, which was then placed in distilled water and subjected to mixing through stirring. In the meantime, ferrous and ferric salts were also dissolved in distilled water. Then, the mixture solutions were mixed together prior to the addition of droplets of sodium hydroxide to elevate the pH to about 10 during heating. The prepared mixture had a 5-to-1 ratio of chitosan to magnetite. The result was a precipitate that was collected and then subjected to successive washing to reduce the pH, and then the formed material was dried to attain the powder composite for use.

### 4.2. Methomyl Oxidation Test

The insecticide C_5_H_10_N_2_O_2_S (“Methomyl”) was used as a simulated aqueous agricultural waste stream. Initially, a 1000 ppm stock solution was prepared, to which successive dilution was applied when various concentrations were required. Hydrogen peroxide (30% *w*/*v*) was used to initiate the catalysis of the chitosan–magnetite reagent. Both reagents were the source of the Fenton reaction. The pH of the insecticide solution was adjusted to the needed values by using sulfuric acid or sodium hydroxide (Sigma-Aldrich, Darmstadt, Germany). Notably, the chemicals used were applied as received from the supplier, without extra purification or treatment.

**Figure 13:**
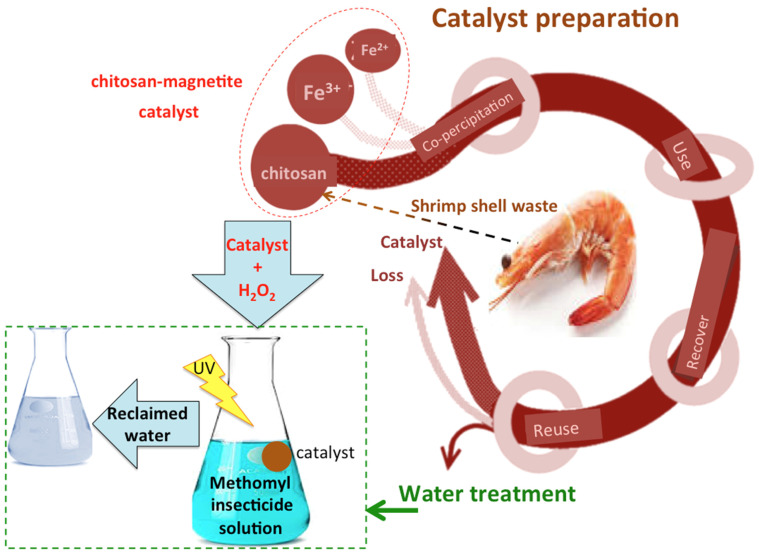
Graphical representation of the catalyst synthesis and wastewater treatment application.

First, 100 mL of “Methomyl” insecticide solution was poured into a glass container in order to investigate the effect of the methomyl concentration on the extent of the Fenton oxidation. Next, both reagents, namely, the chitosan–magnetite composite and H_2_O_2_, were added at the required concentrations to the methomyl solution. Subsequently, the whole mixture was subjected to magnetic stirring to ensure good dispersion under the ultraviolet irradiance. In this regard, a UV lamp (15 W, 230 V/50 Hz, with 253.7 nm wavelength) was used as the ultraviolet source. The operating parameters were investigated, i.e., illumination time (from 10 to 60 min), methomyl loading (50 to 500 mg/L), hydrogen peroxide concentration (100 to 800 mg/L), catalyst (10 to 80 mg/L), and temperature (26 to 60 °C). After specific time intervals, the samples were subjected to spectrophotometric analysis. All of the runs were conducted in triplicate, and the averages were displayed. The solution temperature during the runs was adjusted to the desired values prior to the addition of Fenton’s reagent. The graphical representation of the catalyst preparation and treatment steps is displayed in Figure 13.

### 4.3. Characterization Techniques

The phase structure of the prepared composite was determined through XRD with an X-ray pattern model X-lab Shimadzu X-6000 X-ray diffractometer that worked with a scan step of 0.02°. Also, FTIR analysis was conducted in the wavenumber region of 400–4000 cm^−1^, using a Jasco FTIR-4100 (Hachioji, Tokyo 193-0835, Japan). Composite morphology was explored using scanning electron microscopy (SEM) (model: Quanta FEJ20, Beijing, China) and transmission electron microscopy (TEM) (model: Tecnai G20, FEI, Bellaterra, Barcelona, Spain).

## Figures and Tables

**Table 1:** Parameters of first- and second-order kinetics models for methomyl oxidation by the chitosan–magnetite Fenton system.

Kinetics Model	Parameters	Values			
		T, °C			
		26 °C	40 °C	50 °C	60 °C
Pseudo-first-order ${(C}_{t}=C_{o}-e^{k_{1}t}$)	*k*_1_ (min^−1^)	0.129	0.090	0.101	0.072
	*t*_1/2_ (min)	9.6	6.8	7.7	5.3
	*R* ^2^	0.6	0.59	0.54	0.59
Pseudo-second-order ($\left(\frac{1}{C_{t}}\right)=\left(\frac{1}{C_{0}}\right)-k_{2}t$)	*k*_2_ (L·mg^−1^·min^−1^) × 10^−2^	1.57	0.53	0.31	0.28
	*t*_1/2_ (min)	1.27	3.77	6.45	7.14
	*R* ^2^	0.86	0.94	0.92	0.90

**Table 2:** Thermodynamic parameters for methomyl oxidation by the chitosan–magnetite-based Fenton system.

Thermodynamic Parameters	T/°C			
	26 °C	40 °C	50 °C	60 °C
∆*G*′ (kJ/mol)	83.56	92.50	96.94	100.25
∆*H*′ (kJ/mol)	34.91	34.73	34.65	34.57
∆*S*′ (J/mol K)	−162.70	−180.50	−188.75	−193.18
*Ea* (kJ/mol)	37.39			

## Data Availability

The data presented in this study are available on request from the corresponding author. Please note that there is no restrictions and the data not containing information that could compromise the privacy of research participants.

## References

[1] Abdou K.A., Mohammed A.N., Moselhy W., Farghali A.A. (2018). Assessment of modified rice husk and sawdust as bio-adsorbent for heavy metals removal using nano particles in fish farm. Asian J. Anim. Vet. Adv..

[2] Wulandari I.O., Mardila V.T., Santjojo D.J.D.H., Sabarudin A. (2018). Preparation and Characterization of Chitosan-Coated Fe_3_O_4_ Nanoparticles Using Ex-Situ co-Precipitation Method and Tripolyphosphate/Sulphate as Dual Crosslinkers.

[3] Adesina O.A., Abdulkareem F., Yusuff A.S., Lala M., Okewale A. (2019). Response surface methodology approach to optimization of process parameter for coagulation process of surface water using Moringa oleifera seed. S. Afr. J. Chem. Eng..

[4] Rezgui S., Díez A.M., Monser L., Adhoum N., Pazos M., Sanromán M.A. (2021). ZnFe_2_O_4_-chitosan magnetic beads for the removal of chlordimeform by photo-Fenton process under UVC irradiation. J. Environ. Manag..

[5] Ahmadi M., Behin J., Mahnam A.R. (2016). Kinetics and thermodynamics of peroxydisulfate oxidation of Reactive Yellow 84. J. Saudi Chem. Soc..

[6] Al M.F., Mo’ayyad S., Ahmad S., Mohammad A.-S. (2008). Impact of Fenton and ozone on oxidation of wastewater containing nitroaromatic compounds. J. Environ. Sci..

[7] Argun M.E., Karatas M. (2011). Application of Fenton process for decolorization of reactive black 5 from synthetic wastewater: Kinetics and thermodynamics. Environ. Prog. Sustain. Energy.

[8] Guan X.H., Chen G.H., Shang C. (2015). Re-use of water treatment works sludge to enhance particulate pollutant removal from sewage. Water Res..

[9] Bounab L., Iglesias O., González-Romero E., Pazos M., Sanromán M.Á. (2015). Effective heterogeneous electro-Fenton process of m-cresol with iron loaded actived carbon. RSC Adv..

[10] Clark J.H., Farmer T.J., Herrero-Davila L., Sherwood J. (2016). Circular economy design considerations for research and process development in the chemical sciences. Green Chem..

[11] Di L., Yang H., Xian T., Liu X., Chen X. (2019). Photocatalytic and photo-Fenton catalytic degradation activities of Z-scheme Ag2S/BiFeO3 heterojunction composites under visible-light irradiation. Nanomaterials.

[12] El-Desoky H.S., Ghoneim M.M., El-Sheikh R., Zidan N.M. (2010). Oxidation of Levafix CA reactive azo-dyes in industrial wastewater of textile dyeing by electro-generated Fenton’s reagent. J. Hazard. Mater..

[13] Liu Z., Hosseinzadeh S., Wardenier N., Verheust Y., Chys M., Van Hulle S. (2019). Combining ozone with UV and H_2_O_2_ for the degradation of micropollutants from different origins: Lab-scale analysis and optimization. Environ. Technol..

[14] Gallo-Cordova A., Castro J.J., Winkler E.L., Lima E., Zysler R.D., del Puerto Morales M., Ovejero J.G., Streitwieser D.A. (2021). Improving degradation of real wastewaters with self-heating magnetic nanocatalysts. J. Clean. Prod..

[15] Guedes A.M.F.M., Madeira L.M.P., Boaventura R.A.R., Costa C.A.V. (2003). Fenton oxidation of cork cooking wastewater—Overall kinetic analysis. Water Res..

[16] Hilder M., Winther-Jensen O., Winther-Jensen B., MacFarlane D.R. (2012). Graphene/zinc nano-composites by electrochemical co-deposition. Phys. Chem. Chem. Phys..

[17] Ioannou L.A., Fatta-Kassinos D. (2013). Solar photo-Fenton oxidation against the bioresistant fractions of winery wastewater. J. Environ. Chem. Eng..

[18] Laib S., Rezzaz-Yazid H., Yatmaz H.C., Sadaoui Z. (2021). Low cost effective heterogeneous photo-Fenton catalyst from drinking water treatment residuals for reactive blue 19 degradation: Preparation and characterization. Water Environ. Res..

[19] Li X., Cui J., Pei Y. (2018). Granulation of drinking water treatment residuals as applicable media for phosphorus removal. J. Environ. Manag..

[20] Qu R., Zhang W., Liu N., Zhang Q., Liu Y., Li X., Feng L. (2018). Antioil Ag_3_PO_4_ nanoparticle/polydopamine/Al_2_O_3_ sandwich structure for complex wastewater treatment: Dynamic catalysis under natural light. ACS Sustain. Chem. Eng..

[21] Muthukannan V., Praveen K., Natesan B. (2015). Fabrication and characterization of magnetite/reduced graphene oxide composite incurred from iron ore tailings for high performance application. Mater. Chem. Phys..

[22] Rota F., Cavassi M., Niego D., Gorlani R., Vianelli L., Tatti L., Muntau H. (1996). Mathematical modelling of photomineralization of phenols in aqueous solution, by photocatalytic membranes immobilizing titanium dioxide. Chemosphere.

[23] Oyewo O.A., Adeniyi A., Sithole B.B., Onyango M.S. (2020). Sawdust-based cellulose nanocrystals incorporated with ZnO nanoparticles as efficient adsorption media in the removal of methylene blue dye. ACS Omega.

[24] Pourali P., Behzad M., Arfaeinia H., Ahmadfazeli A., Afshin S., Poureshgh Y., Rashtbari Y. (2021). Removal of acid blue 113 from aqueous solutions using low-cost adsorbent: Adsorption isotherms, thermodynamics, kinetics and regeneration studies. Sep. Sci. Technol..

[25] Rezgui S., Ghazouani M., Bousselmi L., Akrout H. (2022). Efficient treatment for tannery wastewater through sequential electro-Fenton and electrocoagulation processes. J. Environ. Chem. Eng..

[26] Thabet R.H., Fouad M.K., Ali I.A., El Sherbiney S.A., Tony M.A. (2021). Magnetite-based nanoparticles as an efficient hybrid heterogeneous adsorption/oxidation process for reactive textile dye removal from wastewater matrix. Int. J. Environ. Anal. Chem..

[27] Saad R.A., Younes G., El-Dakdouki M.H., Al-Oweini R. (2021). Vanadium-substituted polyoxomolybdates for methylene blue adsorption from aqueous solutions. J. Clust. Sci..

[28] Setyono D., Valiyaveettil S. (2014). Chemically modified sawdust as renewable adsorbent for arsenic removal from water. ACS Sustain. Chem. Eng..

[29] Shaheen T.I., Emam H.E. (2018). Sono-chemical synthesis of cellulose nanocrystals from wood sawdust using acid hydrolysis. Int. J. Biol. Macromol..

[30] Soliman E.M., Ahmed S.A., Fadl A.A. (2020). Adsorptive removal of oil spill from sea water surface using magnetic wood sawdust as a novel nano-composite synthesized via microwave approach. J. Environ. Health Sci. Eng..

[31] Sun J.-H., Sun S.-P., Fan M.-H., Guo H.-Q., Qiao L.-P., Sun R.-X. (2007). A kinetic study on the degradation of p-nitroaniline by Fenton oxidation process. J. Hazard. Mater..

[32] Kaur R., Kaur H. (2021). Solar driven photocatalysis-an efficient method for removal of pesticides from water and wastewater. Biointerface Res. Appl. Chem..

[33] Thabet R.H., Fouad M.K., Sherbiny S.A.E., Tony M.A. (2022). Zero-Waste Approach: Assessment of Aluminum-Based Waste as a Photocatalyst for Industrial Wastewater Treatment Ecology. Int. J. Environ. Res..

[34] Xu Z., Zhang M., Wu J., Liang J., Zhou L., Lǚ B. (2013). Visible light-degradation of azo dye methyl orange using TiO_2_/β-FeOOH as a heterogeneous photo-Fenton-like catalyst. Water Sci. Technol..

[35] Tony M.A., Lin L.-S. (2021). Iron Coated-Sand from Acid Mine Drainage Waste for Being a Catalytic Oxidant Towards Municipal Wastewater Remediation. Int. J. Environ. Res..

[36] Ahamad T., Naushad M., Mousa R.H., Alshehri S.M. (2020). Fabrication of starch-salicylaldehyde based polymer nanocomposite (PNC) for the removal of pollutants from contaminated water. Int. J. Biol. Macromol..

[37] Zhang Y., Klamerth N., Messele S.A., Chelme-Ayala P., El-Din M.G. (2016). Kinetics study on the degradation of a model naphthenic acid by ethylenediamine-N, N’-disuccinic acid-modified Fenton process. J. Hazard. Mater..

[38] Li Y., Jiang J., Fang Y., Cao Z., Chen D., Li N., Xu Q., Lu J. (2018). TiO_2_ nanoparticles anchored onto the metal–organic framework NH2-MIL-88B (Fe) as an adsorptive photocatalyst with enhanced fenton-like degradation of organic pollutants under visible light irradiation. ACS Sustain. Chem. Eng..

[39] Kim I.T., Magasinski A., Jacob K., Yushin G., Tannenbaum R. (2013). Synthesis and electrochemical performance of reduced graphene oxide/maghemite composite anode for lithium ion batteries. Carbon.

[40] Najjar W., Chirchi L., Santos E., Ghorhel A. (2001). Kinetic study of 2-nitrophenol photodegradation on Al-pillared montmorillonite doped with copper. J. Environ. Monit..

[41] Jusin J.W., Aziz M., Sean G.P., Jaafar J. (2016). Preparation and characterization of graphene-based magnetic hybrid nanocomposite. Malays. J. Anal. Sci..

[42] Tony M.A., Eltabey M.M. (2022). End-of-life waste criteria: Synthesis and utilization of Mn–Zn ferrite nanoparticles as a superparamagnetic photocatalyst for synergistic wastewater remediation. Appl. Water Sci..

[43] Pan W., Zhang G., Zheng T., Wang P. (2015). Degradation of p-nitrophenol using CuO/Al 2 O 3 as a Fenton-like catalyst under microwave irradiation. RSC Adv..

[44] Tony M.A. (2022). Pattern, Forms and Bibliometric Analysis for Systematic Study of Silica-Supported Heterogeneous Solar Photocatalyst for Lannate Insecticide Abatement from Aqueous Stream. Arab. J. Sci. Eng..

[45] Gherghel A., Teodosiu C., De Gisi S. (2019). A review on wastewater sludge valorisation and its challenges in the context of circular economy. J. Clean. Prod..

[46] Wang S., Long J., Jiang T., Shao L., Li D., Xie X., Xu F. (2021). Magnetic Fe_3_O_4_/CeO_2_/g-C_3_N_4_ composites with a visible-light response as a high efficiency Fenton photocatalyst to synergistically degrade tetracycline. Sep. Purif. Technol..

